# Validity and reliability of the Brazilian version of the International Consultation on Incontinence Questionnaire Male Sexual Matters Associated with Lower Urinary Tract Symptoms Module*

**DOI:** 10.1590/1518-8345.7481.4545

**Published:** 2025-05-02

**Authors:** André Carlos Santos Ferreira, Dayane Abreu Ribeiro, José Wicto Pereira Borges, M. Graça Pereira, Kátia Santana Freitas, Luciana Regina Ferreira da Mata

**Affiliations:** 1 Universidade Federal de Minas Gerais, Escola de Enfermagem, Belo Horizonte, MG, Brazil; 2 Scholarship holder at the Coordenação de Aperfeiçoamento de Pessoal de Nível Superior (CAPES), Brazil; 3 Scholarship holder at the Fundação de Amparo à Pesquisa do Estado de Minas Gerais (FAPEMIG), Brazil; 4 Universidade Federal do Piauí, Escola de Enfermagem, Floriano, PI, Brazil; 5 Universidade do Minho, Escola de Psicologia, Braga, Minho, Portugal; 6 Universidade Estadual Feira de Santana, Departamento de Saúde, Feira de Santana, BA, Brazil

**Keywords:** Men’s Health, Sexual Dysfunction Physiological, Validation Study, Lower Urinary Tract Symptoms, Patient Reported Outcome Measures, Surveys and Questionnaires

## Abstract

describe the cultural adaptation process of the International Consultation on Incontinence Questionnaire Male Sexual Matters Associated with Lower Urinary Tract Symptoms Module, as well as to analyze validity and reliability of its Brazilian version in men experiencing urinary symptoms.

this cross-sectional study was conducted on 138 men with urinary symptoms who were being followed-up at the Urology outpatient clinic of an Oncology hospital. The internal structure validity evidence was assessed using the four items of the questionnaire, both through exploratory and confirmatory factor analysis. Additionally, reliability was analyzed employing the Cronbach’s α and McDonald’s ω coefficients. The evidence of validity in the relationship with external variables was assessed using Spearman’s correlation with the International Prostate Symptom Score and Male Sexual Quotient.

significant evidence of content validity, response process and a single factor that explained 78% of the variance were detected, with factor loadings ranging between 0.54 and 0.97. Cronbach’s α and McDonald’s ω were close to 0.95, indicating satisfactory reliability. A moderate positive correlation was found between the questionnaire used and the International Prostate Symptom Score (r=0.67), as well as a strong negative correlation with the Male Sexual Quotient (r=-0.84), supporting good evidence of validity in the relationship with external variables.

the Brazilian version of the questionnaire showed strong validity and reliability evidence to assess sexual dysfunctions in the study population.

## Highlights

**(1)** ICIQ-MUTSsex is a valid and reliable assessment questionnaire. **(2)** ICIQ-MUTSsex can be used in research and clinical practice. **(3)** The questionnaire assesses sexual dysfunctions in Brazilian men with urinary symptoms.

## Introduction

Lower Urinary Tract Symptoms (LUTS) represent a broad term encompassing a range of urinary issues, categorized into storage or irritative, voiding or obstructive, and post-voiding symptoms ^( 1 )^. The prevalence of LUTS increases with age, affecting 69% of the men aged at least 40 years old in Brazil ^( 2 )^. The symptoms are associated with sexual difficulties and prostate disorders, thereby impacting men’s Quality of Life (QoL) ^( 3 )^.

For men, sexual identity and erectile function are paramount in maintaining sexual functioning. Sexual Dysfunctions (SDs) are characterized by alterations in one or more phases of the sexual response cycle, which includes the desire, arousal and orgasm stages ^( 4 )^. The prevalence of SDs is directly related to the incidence of LUTS, particularly among men over the age of 40 ^( 2 )^. For instance, a meta-analysis of 24 studies reported that more severe LUTS are associated with increased risk of developing SDs (i.e., increased LUTS severity was correlated with lower levels of erectile function and sexual satisfaction) ^( 3 )^.

From this perspective, considering the relationship between LUTS and SDs and the impact of these conditions on male QoL, the need for health professionals to assess SDs in men with LUTS complaints becomes evident ^( 3 )^. Patient-Reported Outcome Measures (PROMs) offer an alternative for objectively assessing subjective information. These tools support clinical decision-making and enhance understanding of the impact of these conditions on a person’s QoL ^( 5 )^. A literature search to identify PROMs focused on the SD construct revealed 23 instruments, although only two associate SDs with LUTS ^( 6 - 7 )^. In this context, the International Consultation on Incontinence Questionnaire Male Sexual Matters Associated with Lower Urinary Tract Symptoms Module (ICIQ-MLUTSsex) stands out ^( 6 , 8 )^ as a highly recommended instrument (Grade A) ^( 1 )^. Originally developed in 1996 by British scholars ^( 8 )^, ICIQ-MLUTSsex was initially named International Continence Society - Sexual Function Questionnaire (ICSsex). In 1999, the International Consultation on Incontinence Questionnaire (ICIQ) project, initiated by the International Continence Society (ICS), aimed to develop and validate PROMs for patients experiencing lower urinary tract dysfunction, vaginal symptoms and lower bowel dysfunction. Upon the project’s launch, the ICSsex questionnaire was renamed to ICIQ-MLUTSsex ^( 1 )^.

ICIQ-MLUTSsex is a short, easy-to-apply and self-applied questionnaire developed to assess SDs in men experiencing LUTS. Although the psychometric properties of this questionnaire have only been reported in its original version ^( 8 )^, it has undergone cross-cultural adaptations in Canada, South Africa, United States, South Korea, Slovakia and Mexico ^( 9 )^, in addition to various clinical studies ^( 10 - 15 )^. There is no known instrument in Brazil to assess SDs in men with LUTS or evaluate the impact of SDs on QoL. Hence, this study aimed at describing the cultural adaptation process of ICIQ-MLUTSsex and at analyzing validity and reliability of the Brazilian version in men with lower urinary tract symptoms.

## Method

### Study design

With due authorization from the ICIQ group, this study was conducted in two stages. In the first one, the methodological study followed the recommendations for cross-cultural adaptations (translation, back-translation, experts’ committee assessment and pre-test steps) ^( 16 )^. The second stage was a cross-sectional observation study focused on the analysis of the psychometric properties. Evidence of content validity, response process, internal structure and relationship with external variables was verified ^( 17 )^.

### Study setting

The study was developed with patients followed-up at the Urology outpatient clinic of an Oncology hospital in Belo Horizonte (state of Minas Gerais, Brazil).

### Period

The data were collected between January and April 2023.

### Population, selection criteria and sample

The study population consisted in men over the age 18 who complained of at least one LUTS ^( 1 )^, followed-up at the Urology outpatient clinic of an Oncology hospital. Men using indwelling bladder catheters were excluded.

The sample size of psychometric studies is estimated based on the number of items, which shows ratios of 20:1 ^( 18 )^. A minimum sample size of 80 participants was necessary because the instrument has four items. A non-probability, sequential sample comprised by 138 men was recruited. Therefore, the number of participants was adequate based on the literature recommendations ^( 18 )^.

### Original instrument

ICIQ-MLUTSsex is a component of a broader questionnaire (ICSmale), developed in the ICS–“BPH” Study ^( 8 )^, which is structured into three subscales that assess urinary symptoms (22 items), their impact on QoL (seven items) and SDs (four items). In this study, the four ICSmale items covering sexual functioning were considered. The ICIQ-MLUTSsex questionnaire includes four items that assess erection, ejaculation, pain during ejaculation and the impact of LUTS on sex life. Each item is answered using a Likert scale with four options ranging from zero (no change) to three (severe symptoms). The total score varies from zero to 12 points, with higher scores denoting more severe sexual issues. Additionally, the questionnaire includes annoyance scales for each item from zero to 10, with zero indicating “no problem” and 10 indicating “a serious problem”. The annoyance scales are not integrated into the total score but indicate the impact of individual symptoms on the patient ^( 6 , 8 )^.

### Cross-cultural adaptation

Initially, approval to use and adapt ICIQ-MLUTSsex was requested to the ICIQ group. The cross-cultural adaptation process followed a standardized method ^( 16 )^, which is considered in the ICIQ translation protocol. ICIQ-MLUTSsex was translated into Brazilian Portuguese by two Brazilian translators fluent in English, resulting in two independent versions (T1 and T2). Subsequently, a bilingual urologist merged both versions into one (T1+T2). This version was back-translated into English by another two Brazilian translators proficient in this language, creating two independent back-translated versions (RT1 and RT2). The ICIQ group reviewed the back-translated versions and suggested three changes: 1) revising the phrasing of items 2b, 3b, 4b and 5b; 2) replacing the Portuguese translations for “hampered” and “affected” in item 5a with a word that more accurately reflects the negative impact of urinary symptoms on sex life; and 3) proposing removing the term “*ativa*” (“active”) from item 5c. However, this term was introduced during the synthesis of translations in the second stage by a translator with a medical degree and a PhD in Urology. Therefore, we decided to retain it to enhance the participants’ understanding. After these modifications, the ICIQ group approved the Brazilian Portuguese version.

Subsequently, a panel comprised by 20 Brazilian experts (a linguist, three urologists, eight psychometricians, six nurses and two sexologists) assessed content validity for clarity, theoretical relevance and practical applicability of the items examined. The Content Validity Ratio (CVR) was calculated for each item, and CVR values equal to or greater than 0.474 were considered acceptable ^( 19 )^. A pre-test was conducted with 39 men experiencing LUTS ^( 16 )^. During the interviews, the participants were asked about item clarity and theoretical relevance, in addition to whether the answer options were clear and easy to understand. In this stage, a cognitive and semi-structured interview was conducted to evaluate the pre-final version. All interviews were individual and in charge of a single trained interviewer. In the last step, the final version of ICIQ-MLUTSsex translated into Brazilian Portuguese was sent to the original developers of the instrument and its psychometric properties were evaluated.

### Data collection

Data on sociodemographic and clinical factors (age, marital status, schooling level, medical diagnosis, LUTS and comorbidities) were collected. The LUTS were evaluated based on storage symptoms (e.g., pollakiuria, nocturia, urgency and urinary incontinence), micturition symptoms (e.g., slow stream, intermittent stream, hesitation, effort during micturition and terminal dribble) and post-micturition symptoms (e.g., post-micturition dribble and feelings of incomplete emptying) ^( 1 )^. Each participant answered the questionnaires individually, in a private setting and with no time constraints.

The International Prostate Symptom Score includes seven questions focusing on urinary symptoms commonly associated with benign prostatic hyperplasia and a separate question concerning QoL as it pertains to these symptoms. Each question is answered using a Likert scale from 0 (Never) to 5 (Almost always or Five times or more). The aggregate IPSS score is the sum of all seven individual scores, ranging from zero to 35. The higher the score, the more severe the symptom ^( 20 - 21 )^.

The Male Sexual Quotient consists of 10 self-report questions, evaluating aspects such as sexual desire and interest (question 1), self-confidence (question 2), erection quality (questions 5–7), ejaculation control (question 8), ability to reach orgasm (question 9) and satisfaction levels for the man (questions 3, 4 and 10) and for the partner (questions 3 and 10). The items are answered using a five-point Likert scale, with 0 indicating “Never” and 5 denoting “Always”. Higher scores indicate better sexual satisfaction. The total score is calculated by adding up the scores obtained in questions 1–10 and multiplying by two, generating a scale ranging from 0 to 100 where the higher the score, the better the sexual performance ^( 22 - 23 )^.

### Data treatment and analysis

The internal structure validity evidence was assessed by means of exploratory factor analysis followed by confirmatory factor analysis. Dimensionality was assessed through Unidimensional Congruence (UniCO) ≥0.95, explained common variance (ECV) ≥0.85 and Mean of Item Residual Absolute Loading (MIREAL) ≤0.30 ^( 24 )^. Quality and effectiveness of the factor estimates were measured by Factor Determinacy Index (FDI) >0.80, Sensitivity Ratio (SR) >0.2 and Expected Percentage of True Differences (EPTD) >90% ^( 24 )^. The evaluation regarding maintenance or exclusion of items was based on the following criteria: saturation of factor loadings; factor loading ≥0.50; evaluation of items with cross-loadings; and communalities (h^2^) ≥0.40; as well as the practical and conceptual relevance of each item according to the construct to be measured. The model fit indices, such as Root Mean Square Error of Approximation (RMSEA) <0.05, content of fit >0.95 and Tucker-Lewis Index >0.95 were measured ^( 25 )^. The Spearman correlation between the ICIQ-MLUTSsex-Brazilian version scores and IPSS and MSQ defined evidence of validity in the relationship with external variables. Correlation coefficients of 0.40–0.69 indicated moderate correlations, and 0.70 or higher indicated strong correlations ^( 26 )^. The two hypotheses formulated were as follows: a) There is a positive correlation between the total score obtained in ICIQ-MLUTSsex-Brazilian version and the IPSS score; and b) There is a negative correlation between the total score obtained in ICIQ-MLUTSsex-Brazilian version and the MSQ score. Reliability was assessed using McDonald’s ω and Cronbach’s α coefficients, with 0.70 considered satisfactory ^( 27 )^. The Factor analysis software (version 10.10.03) and Mplus software (version 7.4) were used for the statistical analysis.

### Ethical aspects

The research project was approved by the Research Ethics Committee of a teaching institution (no. 5,287,948) according to the recommendations set forth in Resolution 466/2012 from the Brazilian Health Council. All participants signed a Free and Informed Consent Form.

## Results

### Content validity evidence

A committee comprised by 20 experts evaluated content validity of ICIQ-MLUTSsex-Brazilian version during the cross-cultural adaptation process and through cognitive interviews with men presenting LUTS. Content validity of ICIQ-MLUTSsex-Brazilian version was considered very good by the experts’ committee. They suggested excluding the Portuguese term for ‘discomfort’ from item 3a due to its CVR of 0.473. Cognitive interviews were conducted with 39 men with LUTS and aged between 33 and 92 years old (median=65; SD=11.07), of different schooling levels, races and marital statuses. The analysis of the cognitive interviews revealed that items 2a (*Você tem ereções* [Do you have erections?]), 3a (*Você tem ejaculação de sêmen* [Do you ejaculate semen?]) and 4a (*Você sente dor durante a ejaculação de sêmen* [Do you feel pain during ejaculation?]) were unclear, which led to including the terms ‘*pênis duro*’ (‘hard penis’) in item 2a, ‘*você goza*’ (‘do you ejaculate’) in item 3a and ‘*ao gozar*’ (‘when you ejaculate’) in item 4a as synonyms for the confusing terms. The final version of ICIQ-MLUTSsex-Brazilian version was sent to the original developers of the instrument, who approved it, and its psychometric properties were evaluated.

### Characteristics of the sample

One hundred and thirty-eight men aged between 27 and 80 took part in the study. most of them (70.3%) had a cancer diagnosis. Table 1 lists patients’ the sociodemographic and clinical characteristics.

**Table 1 -:** Sociodemographic and clinical characteristics of the participants (n = 138). Belo Horizonte, MG, Brazil, 2023

**Characteristics**	
Age (years old), median (minimum-maximum)	61 (27–80)
Marital status, n* (% ^†^ )	
With a partner	110 (79.7)
Schooling, n* (% ^†^ )	
Elementary School	83 (60.2)
High School	47 (34.1)
Higher Education	08 (5.7)
Medical diagnosis, n* (% ^†^ )	
Prostate cancer	67 (48.6)
Benign prostatic hyperplasia	41 (29.7)
Bladder cancer	12 (8.7)
Penile cancer	10 (7.2)
Testicular cancer	03 (2.2)
Kidney cancer	03 (2.2)
Liver cancer	01 (0.7)
Neck cancer	01 (0.7)
Comorbidities, n* (% ^†^ )	
Hypertension	26 (18.9)
Diabetes	27 (19.5)
Hypertension and diabetes	31 (22.4)
Not applicable	54 (39.2)
Lower urinary tract symptoms, n* (% ^†^ )	
Storage + micturition + post-micturition	80 (58.1)
Storage + micturition	47 (34.1)
Storage	6 (4.3)
Storage + post-micturition	2 (1.4)
Micturition	2 (1.4)
Micturition + post-micturition	1 (0.7)

*n = Sample size; ^†^% = Percentage

### Internal structure validity evidence

The validation process showed that the factor analysis for the ICIQ-MLUTSsex-Brazilian version data matrix was appropriate (Kaiser-Meyer-Olkin=0.81; Bartlett’s sphericity test, *p*<0.05; correlation matrix determinant=0.02). As presented in Table 2, the analysis suggested that ICIQ-MLUTSsex-Brazilian version is a unidimensional instrument. The four-item model was deemed adequate to assess unidimensionality indicators, and the UniCO, ECV and MIREAL indices corroborated unidimensionality of ICIQ-MLUTSsex-Brazilian version. The analysis revealed high explained variance (78%) for dimension 1. Considering factor loadings and communalities, no items were excluded, confirming appropriateness of ICIQ-MLUTSsex-Brazilian version questionnaire model.

**Table 2 -:** Exploratory factor and confirmatory factor analyses to assess the dimensional structure of ICIQ-MLUTSsex-Brazilian version. Belo Horizonte, MG, Brazil, 2023

**Items**		**EFA** *		**CFA** ^†^	
		λ ^‡^	h ^2§^	λ ^‡^	***δ*** ^||^
2a	*Você tem ereções (pênis duro)?*	0.97	0.94	0.97	0.05
3a	*Você tem ejaculação de sêmen (você goza)?*	0.92	0.85	0.95	0.08
4a	*Você sente dor durante a ejaculação de* sêmen *(ao gozar)?*	0.54	0.40	0.76	0.41
5a	*O quanto você sente que sua vida sexual foi prejudicada por seus sintomas urinários?*	0.91	0.83	0.94	0.09
Correlation matrix determinant		0.02			
Bartlett’s sphericity test		*p* <0.05			
Kaiser-Meyer-Olkin index		0.81			
χ ^2^ ¶				7.19 ( *p* =0.02)	
RMSEA**				0.13	
CFI ^††^				0.99	
TLI ^‡‡^				0.99	
Unidimensionality					
UniCO ^§§^		0.99			
ECV ^||||^		0.94			
MIREAL ^¶¶^		0.18			

*EFA = Exploratory Factor Analysis; ^†^CFA = Confirmatory Factor Analysis; ^‡^λ = Factor loadings; ^§^h^2^ = Communalities; ^||^ ***δ*** = Residual variance; ^¶^χ^2^ = Chi-square; **RMSEA = Root Mean Square Error of Approximation; ^††^CFI = Comparative Fit Index; ^‡‡^TLI = Tucker-Lewis Index; ^§§^UniCO = Unidimensional Congruence; ^||||^ECV = Explained Common Variance; ^¶¶^MIREAL = Mean of Item Residual Absolute Loading

### Evidence of validity in the relationship with external variables

There was a moderate positive correlation between the total ICIQ-MLUTSsex-Brazilian version and IPSS (r=0.67; p<0.05) and a strong negative correlation between the total ICIQ-MLUTSsex-Brazilian version and MSQ (r=-0.84; p<0.05), indicating that there was a direct relationship between the variables, confirming the hypotheses established.

### Reliability

Reliability was assessed using McDonald’s ω and Cronbach’s α, which yielded results of 0.95 and excellent replicability, as confirmed by the Construct Reliability - Index G H. The FDI, EPTD and SR indices also indicated good factor estimation (Table 3).

**Figure 1 -:**
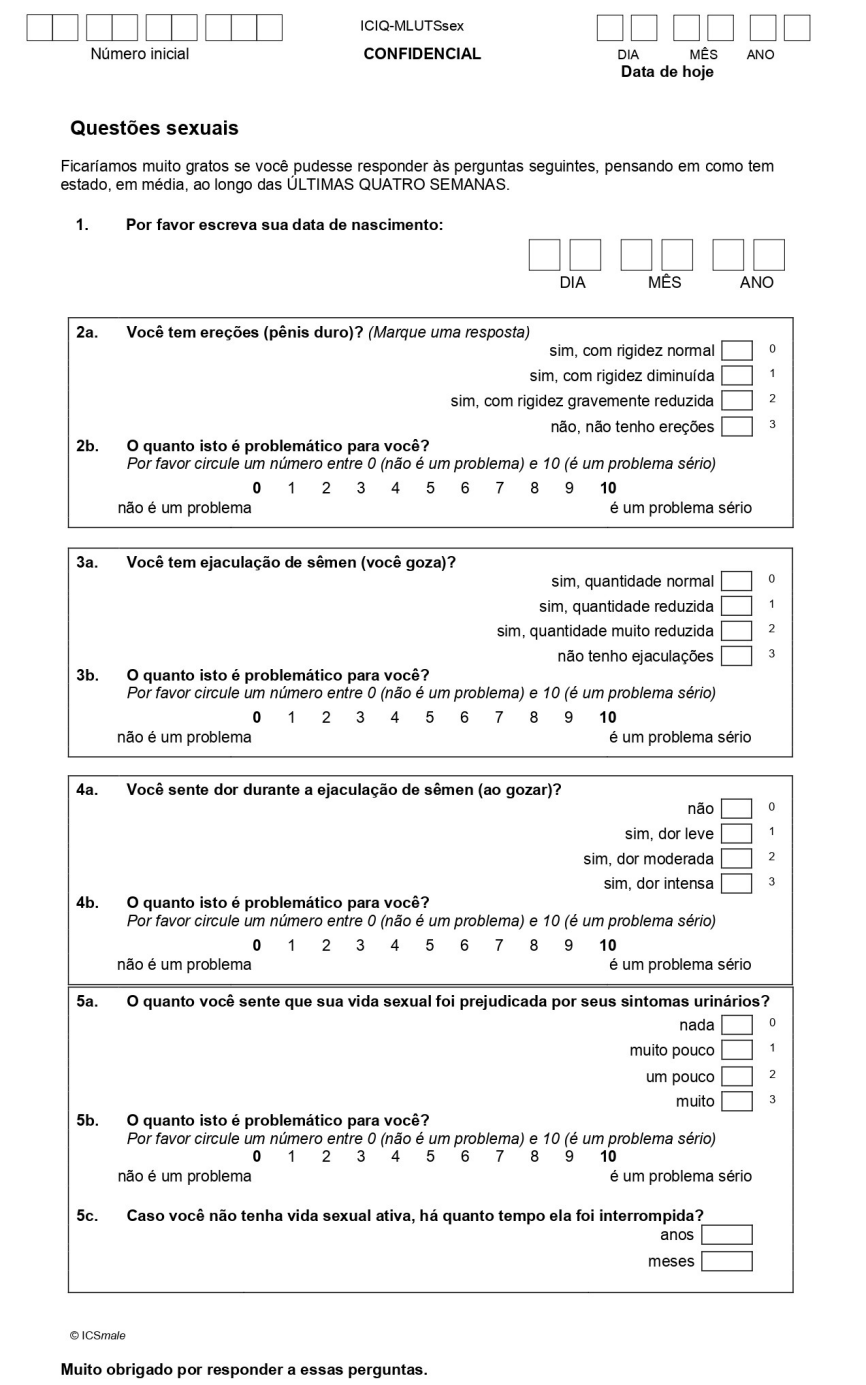
ICIQ-MLUTSsex-Brazilian version. Belo Horizonte, MG, Brazil, 2023

In view of the results obtained, Figure 1 shows the final version of ICIQ-MLUTSsex-Brazilian version.

**Table 3 -:** Reliability, quality and effectiveness indices of ICIQ-MLUTSsex-Brazilian version. Belo Horizonte, MG, Brazil, 2023

**Techniques use** d	**Indices**	**Results**
Reliability	Cronbach’s Alpha	0.95
	McDonald’s Omega	0.95
Quality and efficiency	Construct Reliability - Index G H	0.81
	FDI*	0.98
	EPTD ^†^	100.0% ^‡^
	SR ^§^	41.19

*FDI = Factor Determinacy Index; ^†^EPTD = Expected Percentage of True Differences; ^‡^% = Percentage; ^§^SR = Sensitivity Ratio

## Discussion

The objective of this study was to culturally adapt and analyze validity and reliability of ICIQ-MLUTSsex-Brazilian version in men with LUTS, most of them (70.3%) cancer patients. ICIQ-MLUTSsex-Brazilian version is the first questionnaire adapted to Brazilian Portuguese to evaluate SDs in men with LUTS and the impact of SDs on their QoL ^( 6 )^. In the cross-cultural adaptation process to Brazilian Portuguese, the experts’ committee requested removing the Portuguese term for “discomfort” from item 3a. In the pre-test, they suggested adding the terms “*p*ê*nis duro*”, “*você goza*” and “*ao gozar*” to items 2a, 3a and 4a, respectively. Nonetheless, unlike the Korean ICIQ-MLUTSsex validation study, the answer options for item 2a were not modified ^( 9 )^. The changes made in the adaptation for Brazil were reflected in the positive assessment of the clarity of questions, in the theoretical relevance of the items and in whether the answer options of ICIQ-MLUTSsex-Brazilian version were clear and easy to understand during cognitive interviews.

The cross-cultural adaptation process adhered to specific guidelines ^( 16 )^, resulting in a valid and reliable instrument adapted for self-application in men with LUTS, especially among those with cancer ^( 17 )^. The significant impact of LUTS-related SDs on QoL underscores the need for health professionals to assess the severity of this impact and tailor therapeutic approaches accordingly ^( 28 )^. In this context, PROMs are advocated for individuals with LUTS-related SDs for improved severity assessment and post-therapeutic evaluation ^( 5 )^.

Although the importance of culturally adapting questionnaires is acknowledged, inadequacy of adaptation procedures remains a concern among researchers. Proper adaptation requires careful consideration of several factors such as cultural, idiomatic, linguistic and contextual elements related to the instrument’s translation, which is paramount ^( 16 )^.

In the original version of ICIQ-MLUTSsex ^( 8 )^, the triangular validation model outlined by the Classical Test Theory (content, criterion and construct validity) was employed ^( 29 )^. To establish the questionnaire’s capability to measure SDs, the creators assessed its construct validity by correlating the item scores to symptoms reported by the participants, albeit without examining its internal structure ^( 8 )^.

The current measurement theory underscores that any instrument designed to measure a construct should possess an internal structure that reflects the empirical reality of that construct. This is crucial for interpreting the scores derived from the instrument ^( 30 )^. Therefore, the internal structure validity evidence signifies the extent to which the construct theory underpins and elucidates the test results. This evidence can be established through EFAs and CFAs ^( 31 )^.

ICIQ-MLUTSsex-Brazilian version was evaluated for internal structure evidence using modern psychometrics. This approach is considered the gold standard in test development, validation and utilization, ensuring that tests are equitable, reliable and valid ^( 17 )^. For the validity assessment evidence, a polychoric correlation matrix was employed due to the ordinal nature of the data ^( 24 )^. The matrix yielded favorable results, as supported by the satisfactory adequacy indices. ICIQ-MLUTSsex-Brazilian version was unidimensional, with high factor loadings and adequate communalities ^( 18 )^. Items 2a, 3a and 5a presented factor loadings close to 1 and high commonality, indicating that they may be measuring aspects of the construct that are very similar. This suggests that these items should be the focus of future studies with diverse samples to confirm their behavior. Through the EFA, we identified common variance proportion that was satisfactory and represented 78% of the explained variance ^( 32 )^, which supports its consistency and robustness for measuring the SD construct in men with LUTS.

As for the factor loadings, the values were acceptable given the sample size (0.50 for samples between 120 and 150 participants) ^( 18 )^. The communality values of the items were considered indicators of the variability explained by the factors, providing an assessment of the quality of the single-factor model ^( 18 )^. Moreover, the UniCO, ECV and MIREAL indices confirmed unidimensionality of the instrument, and replicability was confirmed through the Construct Reliability - Index G H. The CFA corroborated the unidimensional model of ICIQ-MLUTSsex-Brazilian version with four items with significant factor loadings, acceptable residual variances and sufficient adjustment indices, showing that the module for SDs has an internal structure that represents the construct. While the RMSEA fit index exceeded 0.05, this discrepancy was attributed to the insufficient sample size of this study for accurately estimating this index ^( 18 , 25 )^. These results align with the theory underpinning the items in the original instrument ^( 8 )^, which is based on the four main symptoms representing LUTS-associated SDs ^( 6 )^.

Validity varies in measuring instruments, as psychometric properties are not static but depend on the interaction between the population in which the instrument is used and the circumstances of its use ^( 33 )^. Therefore, the internal structure analysis suggested that the four questions formulated in the original study ^( 8 )^ were effective in measuring and explaining these symptoms in men with LUTS.

As for reliability, Cronbach’s α was higher that of the original study sample ^( 8 )^ and, when coupled with McDonald’s ω ^( 17 , 27 )^, confirmed reliability of ICIQ-MLUTSsex-Brazilian version. This means that the items have a consistent interrelation for evaluating the SDs in men with LUTS construct and that the respondents’ scores do not change as much over time.

The evidence of validity in the relationship with external variables verified a direct link between prostate symptoms and SDs. These findings are consistent with the existing literature ^( 2 - 3 )^, supporting the notion that prostate symptoms are predictive of SDs.

Despite the promising results, one of the limitations of this study is that the pre-test and empirical validation stages were conducted in a single Urology outpatient clinic of an Oncology hospital, with over half of the sample represented by men with cancer. Therefore, future studies should extend their sample to verify validity of the findings across different Brazilian regions and among different populations within the field of urological diseases. Another constraint was that the original study did not allow for a direct comparison with the Brazilian version of the instrument, primarily because the variables used to validate the relationship with external variables were derived from uroflowmetry exams ^( 8 )^. The absence of uroflowmetry data in the medical records precluded replicating the original study evidence analysis, which involved comparing findings to frequency-volume diaries and uroflowmetry results ^( 8 )^.

ICIQ-MLUTSsex-Brazilian version contributes significant theoretical value to the cross-cultural adaptation process of this instrument. This version stands out because it was subjected to robust psychometric analyses, which confirmed the presence of an internal structure supporting the interpretation of its items. Unlike this comprehensive approach, the adaptations for South Africa, Canada, United States, Korea, Mexico and Slovakia were part of a multicenter study involving a modest sample comprised by 30 participants. This study focused primarily on linguistic aspects to ensure item comprehension ^( 9 )^, without delving into any verification of other types of validity evidence necessary for accurately measuring the construct.

Using this instrument will allow for new interpretative horizons on the SDs of people with LUTS in Brazil. Its results might support nurses’ care actions in different health care scenarios, as well as direct actions towards more effective strategies for reducing the impact of SDs on men’s quality of life. Additionally, this research contributes robust techniques and was based on contemporary psychometric recommendations.

## Conclusion

The current study establishes that ICIQ-MLUTSsex-Brazilian version for men with LUTS, especially among men with cancer, presents both strong validity and reliability evidence. This marks a significant advancement by providing a novel research avenue for the initial assessment of SDs in Brazilian men with LUTS, as it fills a gap left by the absence of other validated tools linking LUTS and SDs. This PROM is anticipated to be highly beneficial in future research and clinical practice.
