# Supplementary material for: Validity and reliability of the Brazilian version of the International Consultation on Incontinence Questionnaire Male Sexual Matters Associated with Lower Urinary Tract Symptoms Module
Source: Rev Lat Am Enfermagem. 2025 May 2;33:e4545. doi: 10.1590/1518-8345.7481.4545 (PMC12052310; doi:10.1590/1518-8345.7481.4545)
Supplement: Translation

# Translation: Validade e confiabilidade da versão brasileira do módulo Problemas Sexuais Masculinos Associados a Sintomas do Trato Urinário Inferior do questionário Consulta Internacional sobre Incontinência*

**DOI:** 10.1590/1518-8345.7481.4546

**Authors:** André Carlos Santos Ferreira, Dayane Abreu Ribeiro, José Wicto Pereira Borges, M. Graça Pereira, Kátia Santana Freitas, Luciana Regina Ferreira da Mata

**Affiliations:** 1 Universidade Federal de Minas Gerais, Escola de Enfermagem, Belo Horizonte, MG, Brasil; 2 Bolsista da Coordenação de Aperfeiçoamento de Pessoal de Nível Superior (CAPES), Brasil; 3 Bolsista da Fundação de Amparo à Pesquisa do Estado de Minas Gerais (FAPEMIG), Brasil; 4 Universidade Federal do Piauí, Escola de Enfermagem, Floriano, PI, Brasil; 5 Universidade do Minho, Escola de Psicologia, Braga, Minho, Portugal; 6 Universidade Estadual Feira de Santana, Departamento de Saúde, Feira de Santana, BA, Brasil

**Keywords:** Saúde do Homem, Disfunções Sexuais Fisiológicas, Estudo de Validação, Sintomas do Trato Urinário Inferior, Medidas de Resultados Relatados pelo Paciente, Inquéritos e Questionários

## Abstract

descrever o processo de adaptação cultural do módulo Problemas Sexuais Masculinos Associados a Sintomas do Trato Urinário Inferior do questionário Consulta Internacional sobre Incontinência, bem como analisar a validade e confiabilidade de sua versão brasileira em homens com sintomas urinários.

este estudo transversal foi realizado com 138 homens com sintomas urinários acompanhados no ambulatório de Urologia de um hospital especializado em Oncologia. As evidências de validade da estrutura interna foram avaliadas por meio dos quatro itens do questionário, mediante análise fatorial exploratória e confirmatória. Além disso, a confiabilidade foi analisada pelos coeficientes α de Cronbach e ω de McDonald. As evidências de validade na relação com variáveis externas foram avaliadas por meio da correlação de Spearman com o *International Prostate Symptom Score* (IPSS) e o Quociente Sexual Masculino (*Male Sexual Quotient,*MSQ).

foram detectadas evidências significativas de validade de conteúdo, processo de resposta e um único fator que explicou 78% da variância, com cargas fatoriais que variaram entre 0,54 e 0,97. Os coeficientes α de Cronbach e ω de McDonald atingiram valores próximos de 0,95, indicando nível de confiabilidade satisfatório. Foi encontrada correlação positiva moderada entre o questionário utilizado e o *International Prostate Symptom Score* (r=0,67), bem como forte correlação negativa com o *Male Sexual Quotient* (r=-0,84), indicando boas evidências de validade na relação com variáveis externas.

a versão brasileira do questionário apresentou sólidas evidências de validade e confiabilidade para avaliar disfunções sexuais na população estudada.

## Destaques

**(1)** O ICIQ-MUTSsex é um questionário de avaliação válido e confiável. **(2)** O ICIQ-MUTSsex pode ser utilizado em pesquisa e na prática clínica. **(3)** O questionário avalia disfunções sexuais em brasileiros que sofrem sintomas urinários.

## Introdução

Os sintomas do trato urinário inferior (STUI) representam um termo geral que abrange vários problemas urinários, categorizados em sintomas de armazenamento ou irritação, sintomas miccionais ou obstrutivos e sintomas pós-miccionais ^( 1 )^. A prevalência de STUI aumenta com a idade e afeta 69% dos homens com pelo menos 40 anos de idade no Brasil ^( 2 )^. Os sintomas estão associados a dificuldades sexuais e distúrbios da próstata, afetando a Qualidade de Vida (QV) masculina ^( 3 )^.

Para os homens, a identidade sexual e a disfunção eréctil são essenciais para manter a sua vida sexual. As Disfunções Sexuais (DS) são caracterizadas por alterações em pelo menos uma das etapas do ciclo de resposta sexual, que inclui as fases de desejo, excitação e orgasmo ^( 4 )^. A prevalência das DS está diretamente relacionada à incidência de STUI, principalmente em homens com mais de 40 anos ^( 2 )^. Por exemplo, uma metanálise de 24 estudos relatou que sintomas mais graves de STUI estão associados a um risco maior de desenvolver diversas DS (ou seja, sintomas mais graves de STUI foram correlacionados com níveis mais baixos de função erétil e satisfação sexual) ^( 3 )^.

Sob esse ponto de vista, considerando a relação entre sintomas de STUI e DS e o efeito dessas doenças na QV masculina, torna-se evidente a necessidade de os profissionais de saúde avaliarem DS em homens com problemas relacionados aos sintomas de STUI ^( 3 )^. As medidas de resultados relatados pelo paciente (*Patient-Reported Outcome Measures*, PROMs) oferecem uma alternativa para avaliar objetivamente informações subjetivas. Essas ferramentas apoiam a tomada de decisões clínicas e permitem uma melhor compreensão do efeito dessas doenças na QV de uma pessoa ^( 5 )^. Em busca realizada na literatura para identificar medidas de PROMs focadas no construto de DS, foram encontrados 23 instrumentos, embora apenas dois deles associassem DS a STUI ^( 6 - 7 )^. Nesse contexto, destaca-se o Questionário Internacional *de Consulta* sobre Incontinência (*International Consultation on Incontinence Questionnaire Male Sexual Matters Associated with Lower Urinary Tract Symptoms Module*, ICIQ-MLUTSsex) ^( 6 , 8 )^ como um instrumento altamente recomendado (Nível A) ^( 1 )^. Originalmente desenvolvido em 1996 por acadêmicos britânicos ^( 8 )^, o nome inicial do ICIQ-MLUTSsex foi *International Continence Society - Sexual Function Questionnaire* (ICSsex). Em 1999, iniciado pela Sociedade Internacional de Continência (*International Continence Society*, ICS), o projeto do Questionário de Consulta Internacional sobre Incontinência (*International Consultation on Incontinence Questionnaire, ICIQ*) teve como objetivo desenvolver e validar medidas PROMs para pacientes que sofrem de disfunção do trato urinário inferior, sintomas vaginais e disfunção intestinal inferior. Quando o projeto foi lançado, o nome do questionário ICSsex foi alterado para ICIQ-MLUTSsex ^( 1 )^.

O ICIQ-MLUTSsex é um questionário breve, simples e autoaplicável que foi desenvolvido para avaliar diversas DS em homens com STUI. Embora suas propriedades psicométricas tenham sido relatadas apenas em sua versão original ^( 8 )^, foi realizada a adaptação transcultural no Canadá, África do Sul, Estados Unidos, Coreia do Sul, Eslováquia e México ^( 9 )^, além de ter sido objeto de diversos estudos clínicos ^( 10 - 15 )^. No Brasil, não há referência de instrumentos para avaliar DS em homens com STUI ou para avaliar o efeito de DS na QV. Portanto, o objetivo deste estudo foi descrever o processo de adaptação cultural do ICIQ-MLUTSsex e analisar a validade e confiabilidade de sua versão brasileira em homens com sintomas do trato urinário inferior.

## Método

### Design do estudo

Com a devida autorização do grupo ICIQ, este estudo foi realizado em duas etapas. Na primeira, o estudo metodológico seguiu as recomendações para a realização de adaptações transculturais (tradução, retrotradução, avaliação por comitê de especialistas e fases para pré-testes) ^( 16 )^. A segunda etapa consistiu em um estudo observacional e transversal focado na análise de propriedades psicométricas. Foram verificadas evidências de validade de conteúdo, processo de resposta, estrutura interna e relação com variáveis externas ^( 17 )^.

### Local do estudo

O estudo foi realizado com pacientes em acompanhamento no ambulatório de Urologia de um hospital especializado em Oncologia de Belo Horizonte (MG).

### Período

Os dados foram coletados entre janeiro e abril de 2024.

### População, critérios de elegibilidade e amostra

A população do estudo foi composta por homens com 18 anos ou mais que relataram sofrer de pelo menos um STUI ^( 1 )^, em acompanhamento no ambulatório de Urologia de um hospital especializado em Oncologia. Foram excluídos homens com cateter urinário de demora.

Em estudos psicométricos, o tamanho da amostra é estimado com base no número de itens, que apresenta proporções de 20:1 ^( 18 )^. Foi necessária uma amostra mínima de 80 participantes pois o instrumento possui quatro itens. Foi recrutada amostragem não probabilística e sequencial composta por 138 homens. Consequentemente, o número de participantes foi adequado com base nas recomendações indicadas na literatura ^( 18 )^.

### Instrumento original

O ICIQ-MLUTSsex faz parte de um questionário mais extenso (*ICS–“BPH” Study*) ^( 8 )^, cuja estrutura conta com três subescalas que avaliam sintomas urinários (22 itens), seu efeito na QV (sete itens) e DS (quatro itens). Neste estudo foram considerados os quatro itens do ICSmale que abrangem o funcionamento sexual. O questionário ICIQ-MLUTSsex inclui quatro itens que avaliam a ereção, ejaculação, dor durante a ejaculação e efeito dos STUI na QV Cada item é respondido por meio de uma escala Likert com quatro opções que variam de zero (sem alteração) a três (sintomas graves). O escore total varia de zero a 12 pontos, sendo que valores mais elevados indicam problemas sexuais mais graves. Além disso, o questionário inclui escalas de desconforto para cada item (de zero a 10), na qual zero indica “nenhum problema” e 10 representa “um problema grave”. As escalas de desconforto não estão integradas no escore final, mas indicam o efeito de cada sintoma no paciente ^( 6 , 8 )^.

### Adaptação transcultural

Como primeira medida, buscou-se a aprovação do uso e adaptação do ICIQ-MLUTSsex ao grupo ICIQ. No processo de adaptação transcultural foi seguido um método padronizado ^( 16 )^, que é considerado no protocolo de tradução do ICIQ. Os responsáveis pela tradução do ICIQ-MLUTSsex para o português brasileiro foram dois tradutores brasileiros fluentes em inglês, gerando assim duas versões independentes (T1 e T2). Posteriormente, um urologista bilíngue combinou as duas versões em uma só (T1+T2). Outros dois tradutores brasileiros com perfeito domínio do inglês retrotraduziram esta versão para esse idioma, gerando duas versões retrotraduzidas independentes (RT1 e RT2). O grupo ICIQ revisou as versões retrotraduzidas e sugeriu três alterações: 1) reformulação dos itens 2b, 3b, 4b e 5b; 2) substituir a tradução portuguesa correspondente a “*hampered*” e “*affected*” no item 5a por uma palavra que reflita com maior precisão o efeito negativo dos sintomas urinários na vida sexual; e 3) propor a retirada do termo “*ativa*” do item 5c. Porém, quem introduziu esse termo durante a síntese das traduções na segunda etapa foi um tradutor formado em medicina e doutor em Urologia. Consequentemente, decidimos mantê-lo para que os participantes pudessem compreender melhor o conteúdo. Após essas modificações, o grupo ICIQ aprovou a versão em português brasileiro.

Posteriormente, um painel composto por 20 especialistas brasileiros (um especialista em linguística, três urologistas, oito especialistas em psicometria, seis enfermeiros e dois sexólogos) avaliou a validade do conteúdo quanto à clareza, relevância teórica e facilidade de aplicação prática dos itens examinado. Foi calculada a razão de validade de conteúdo (CVR) para cada item; Valores de CVR iguais ou superiores a 0,474 foram considerados aceitáveis ^( 19 )^. Um pré-teste foi realizado em 39 homens portadores de STUI ^( 16 )^. Durante as entrevistas, os participantes foram questionados sobre a clareza e relevância teórica dos itens, bem como se as opções de resposta eram claras e de fácil compreensão. Nesta etapa foram realizadas entrevistas cognitivas e semiestruturadas para avaliação da versão pré-final. Todas as entrevistas foram individuais e conduzidas por um único entrevistador devidamente treinado. Na última etapa, a versão do ICIQ-MLUTSsex traduzida para o português brasileiro foi enviada aos desenvolvedores originais do instrumento e suas propriedades psicométricas foram avaliadas.

### Coleta de dados

Foram coletados dados sobre fatores sociodemográficos e clínicos (idade, estado civil, nível educacional, diagnóstico médico, sintomas de STUI e comorbidades). Os STUI foram avaliados com base nos sintomas de armazenamento (ex., polaciúria, noctúria, urgência e incontinência urinária), sintomas de micção (ex., jato lento de urina, jato de urina intermitente, hesitação durante a micção, esforço durante a micção e gotejamento final) e sintomas pós-miccionais (ex., gotejamento após urinar e sensação de esvaziamento incompleto) ^( 1 )^. Cada participante respondeu aos questionários individualmente, em local privativo e sem limite de tempo.

O *International Prostate Symptom Score* inclui sete perguntas focadas em sintomas urinários comumente associados à hiperplasia prostática benigna, bem como uma pergunta separada sobre QV em relação a esses sintomas. Cada pergunta é respondida através de uma escala Likert de 0 (Nunca) a 5 (Quase sempre ou Pelo menos cinco vezes). A pontuação cumulativa do IPSS é a soma dos sete escores individuais e varia de zero a 35. Quanto maior a pontuação, mais grave é o sintoma ^( 20 - 21 )^.

O *Male Sexual Quotient* é composto por 10 perguntas autorrelatadas que avaliam aspectos como desejo e interesse sexual (pergunta 1), autoconfiança (pergunta 2), qualidade da ereção (perguntas 5 a 7), controle da ejaculação (pergunta 8), capacidade de alcançar orgasmo (pergunta 9) e nível de satisfação, tanto para o homem (perguntas 3, 4 e 10) quanto para o casal (perguntas 3 e 10). Os itens são respondidos em uma escala Likert de cinco pontos, na qual 0 indica “Nunca” e 5 “Sempre”. Escores mais altos indicam maior satisfação sexual. O escore total é calculado somando os valores obtidos nas perguntas 1 a 10 e multiplicando-os por dois. Dessa forma, gera-se uma escala de 0 a 100 em que quanto maior a pontuação, melhor o desempenho sexual ^( 22 - 23 )^.

### Processamento e análise de dados

As evidências de validade da estrutura interna foram avaliadas por meio de análise fatorial exploratória, seguida de análise fatorial confirmatória. A dimensionalidade foi avaliada pela Congruência Unidimensional (*Unidimensional COngruence*, UniCO) ≥0,95, Variância Comum Explicada (VCE) ≥0,85 e pela *Mean of Item Residual Absolute Loading* (MIREAL) ≤0,30 ^( 24 )^. A qualidade e efetividade das estimativas fatoriais foram medidas com Índice de Determinação Fatorial (*Factor Determinacy Index*, FDI) >0,80, Razão de Sensibilidade (RS) >0,2 e *Expected Percentage of True Differences* (EPTD) >90% ^( 24 )^. A avaliação quanto à manutenção ou exclusão de itens baseou-se nos seguintes critérios: saturação das cargas fatoriais; carga fatorial ≥0,50; avaliação de itens com cargas transversais; e comunalidades (h^2^) ≥0,40; bem como a relevância prática e conceitual de cada item de acordo com o construto a ser mensurado. Foram medidos índices de ajuste do modelo, como *Root Mean Square Error of Approximation* (RMSEA) <0,05, conteúdo de ajuste >0,95 e índice de Tucker-Lewis >0,95 ^( 25 )^. A correlação de Spearman entre o ICIQ-MLUTSsex-Versão Brasileira e os escores do IPSS e do MSQ definiu evidências de validade na relação com variáveis externas. Coeficientes de correlação de 0,40 a 0,69 indicaram correlações moderadas, enquanto valores de pelo menos 0,70 indicaram correlações fortes ^( 26 )^. Foram formuladas duas hipóteses: a) Existe correlação positiva entre a pontuação total obtida no ICIQ-MLUTSsex-Versão Brasileira e o score do IPSS; e b) Existe correlação negativa entre a pontuação total obtida no ICIQ-MLUTSsex-Versão Brasileira e a pontuação do MSQ. A confiabilidade foi avaliada pelos coeficientes ω de McDonald e α de Cronbach, considerando 0,70 como valor satisfatório ^( 27 )^. Os programas Factor (versão 10.10.03) e Mplus (versão 7.4) foram utilizados para análise estatística.

### Aspectos éticos

O projeto de pesquisa foi aprovado pelo Comitê de Ética em Pesquisa de uma instituição de ensino (nº 5.287.948) de acordo com as recomendações estipuladas na Resolução 466/2012 do Conselho Nacional de Saúde. Todos os participantes assinaram o Termo de Consentimento Livre e Esclarecido.

## Resultados

### Evidência de validade de conteúdo

Um comitê composto por 20 especialistas avaliou a validade de conteúdo do ICIQ-MLUTSsex-Versão Brasileira durante o processo de adaptação transcultural e por meio de entrevistas cognitivas com homens com sintomas de STUI. O comitê de especialistas considerou a validade de conteúdo do ICIQ-MLUTSsex-Versão Brasileira muito boa. Sugeriram excluir a tradução de ‘*discomfort*’ para o português do item 3a devido a sua CVR de 0,473. Foram realizadas entrevistas cognitivas com 39 homens com sintomas de STUI, com idade entre 33 e 92 anos (mediana=65; DP=11,07) e com diferentes níveis de escolaridade, raças e estados civis. A análise das entrevistas cognitivas revelou que os itens 2 (Você tem ereções?), 3 (Você tem ejaculação de sêmen) e 4 (Você sente dor durante a ejaculação de sêmen) não eram claros; o que levou à inclusão dos termos ‘pênis duro’ no item 2a, ‘você goza’ no item 3a e ‘ao gozar’ no item 4a como sinônimos das palavras confusas. A versão final do ICIQ-MLUTSsex - Versão Brasileira foi enviada aos desenvolvedores originais do instrumento (que o aprovaram) e suas propriedades psicométricas foram avaliadas.

### Características da amostra

Participaram do estudo 138 homens com idade entre 27 e 80 anos, a maioria (70,3%) com diagnóstico de câncer. A Tabela 1 mostra as características sociodemográficas e clínicas dos pacientes.

**Tabela 1 -:** Características sociodemográficas e clínicas dos participantes (n = 138). Belo Horizonte, MG, Brasil, 2023

**Características**	
Idade (anos), mediana (mínimo-máximo)	61 (27-80)
Estado civil, n* (% ^†^ )	
Com parceira(o)	110 (79,7)
Nível educacional, n* (% ^†^ )	
Ensino fundamental	83 (60,2)
Ensino médio	47 (34,1)
Ensino superior	08 (5,7)
Diagnóstico médico, n* (% ^†^ )	
Câncer de próstata	67 (48,6)
Hiperplasia prostática benigna	41 (29,7)
Câncer de bexiga	12 (8,7)
Câncer de pênis	10 (7,2)
Câncer testicular	03 (2,2)
Câncer renal	03 (2,2)
Câncer de fígado	01 (0,7)
Câncer de pescoço	01 (0,7)
Comorbidades, n* (% ^†^ )	
Hipertensão	26 (18,9)
Diabetes	27 (19,5)
Hipertensão e diabetes	31 (22,4)
Não aplicável	54 (39,2)
Sintomas do trato urinário inferior, n* (% ^†^ )	
Armazenamento + micção + pós-micção	80 (58,1)
Armazenamento + micção	47 (34,1)
Armazenamento	6 (4,3)
Armazenamento + pós-vazio	2 (1,4)
Micção	2 (1,4)
Micção + pós-micção	1 (0,7)

*n = Tamanho da amostra; ^†^% = Porcentagem

### Evidência de validade da estrutura interna

No processo de validação, demonstrou-se que a análise fatorial para a matriz de dados do ICIQ-MLUTSsex-Versão Brasileira foi adequada (Kaiser-Meyer-Olkin=0,81; teste de esfericidade de Bartlett, *p*<0,05; determinante da matriz de correlação=0,02). Conforme apresentado na Tabela 2, a análise sugeriu que o ICIQ-MLUTSsex-Versão Brasileira é um instrumento unidimensional. O modelo com quatro itens foi considerado adequado para avaliar indicadores de unidimensionalidade. Além disso, os índices UniCO, VCE e MIREAL corroboraram a unidimensionalidade do ICIQ-MLUTSsex-Versão Brasileira. A análise revelou alto percentual de variância explicada (78%) para a dimensão 1. Considerando cargas fatoriais e comunalidades, nenhum item foi excluído, confirmando assim a adequação do modelo do questionário ICIQ-MLUTSsex-Versão Brasileira.

**Tabela 2 -:** Análises fatoriais exploratórias e confirmatórias para avaliação da estrutura dimensional do ICIQ-MLUTSsex-Versão Brasileira. Belo Horizonte, MG, Brasil, 2023

**Item**		**AFE** *		**AFC** ^†^	
		λ ^‡^	h ^2§^	λ ^‡^	***δ*** ^||^
2a	Você tem ereções (pênis duro)?	0,97	0,94	0,97	0,05
3a	Você tem ejaculação de sêmen (você goza)?	0,92	0,85	0,95	0,08
4a	Você sente dor durante a ejaculação do sêmen (ao gozar)?	0,54	0,40	0,76	0,41
5a	O quanto você sente que sua vida sexual foi prejudicada pelos seus sintomas urinários?	0,91	0,83	0,94	0,09
Determinante da matriz de correlação		0,02			
Teste de esfericidade de Bartlett		*p* <0,05			
Índice Kaiser-Meyer-Olkin		0,81			
χ ^2¶^				7,19 ( *p* =0,02)	
RMSEA**				0,13	
IAC ^††^				0,99	
ITL ^‡‡^				0,99	
Unidimensionalidade					
UniCO ^§§^		0,99			
VCE ^||||^		0,94			
MIREAL ^¶¶^		0,18			

*AFE = Análise Fatorial Exploratória; ^†^AFC = Análise Fatorial Confirmatória; ^‡^λ = Cargas fatoriais; ^§^h^2^ = Comunalidades; ^||^ ***δ =*** Variância residual; ^¶^χ^2^ = Qui quadrado; **RMSEA =*Root Mean Square Error of Approximation*; ^††^IAC = Índice de Ajuste Comparativo; ^‡‡^ITL = Índice de Tucker-Lewis; ^§§^UniCO = Congruência Unidimensional; ^||||^VCE = Variância Comum Explicada; ^¶¶^MIREAL = *Mean of Item Residual Absolute Loading*

### Evidência de validade na relação com variáveis externas

Foi registrada uma correlação positiva moderada entre todo o instrumento ICIQ-MLUTSsex-Versão Brasileira e o IPSS (r=0,67; p<0,05) e uma correlação negativa forte entre todo o instrumento ICIQ-MLUTSsex-Versão Brasileira e o MSQ (r=-0,84; p<0,05). Isso indica que houve relação direta entre as variáveis, confirmando assim as hipóteses estabelecidas.

### Confiabilidade

A confiabilidade foi avaliada pelos coeficientes ω de McDonald e α de Cronbach, obtendo resultados de 0,95 e excelente replicabilidade, conforme confirmado pelo *Construct Reliability - Index G H*. Os índices FDI, EPTD e RS também indicaram boa estimativa dos fatores (Tabela 3).

Diante dos resultados obtidos, a Figura 1 apresenta a versão final do ICIQ-MLUTSsex-Versão Brasileira.

**Tabela 3 -:** Índices de confiabilidade, qualidade e efetividade correspondentes ao ICIQ-MLUTSsex-versão brasileira. Belo Horizonte, MG, Brasil, 2023

**Técnicas utilizadas**	Índices	**Resultados**
Confiabilidade	Alfa de Cronbach	0,95
	Ômega de McDonald	0,95
Qualidade e eficiência	*Construct Reliability - Index G H*	0,81
	FDI*	0,98
	EPTD ^†^	100,0% ^‡^
	RS ^§^	41.19

*FDI = *Factor Determinacy Index*; ^†^EPTD = *Expected Percentage of True Differences*; ^‡^% = Porcentagem; ^§^RS = Razão de Sensibilidade

**Figura 1 -:** ICIQ-MLUTSsex-Versão Brasileira. Belo Horizonte, MG, Brasil, 2023

## Discussão

O objetivo deste estudo foi adaptar culturalmente o ICIQ-MLUTSsex-Versão Brasileira e analisar sua validade e confiabilidade em homens com sintomas de STUI, em sua maioria (70,3%) pacientes com câncer. O ICIQ-MLUTSsex-Versão Brasileira é o primeiro questionário adaptado para o português brasileiro para avaliar DS em homens com STUI e o efeito das DS na sua QV ^( 6 )^. No processo de adaptação transcultural para o português brasileiro, o comitê de especialistas solicitou a retirada do termo português correspondente a “*discomfort*” do item 3a. No pré-teste, sugeriram acrescentar os termos “pênis duro”, “você goza” e “ao gozar” aos itens 2a, 3a e 4a, respectivamente. Entretanto, diferentemente do estudo de validação coreano do ICIQ-MLUTSsex, as opções de resposta para o item 2a não foram modificadas ^( 9 )^. As mudanças realizadas na adaptação para o Brasil refletiram-se na avaliação positiva sobre a clareza das perguntas e a relevância teórica dos itens, considerando-se a definição das opções de respostas do ICIQ-MLUTSsex-Versão Brasileira como claras e de fácil compreensão durante as entrevistas cognitivas.

No processo de adaptação transcultural foram respeitadas orientações específicas ^( 16 )^. Isto resultou em um instrumento válido e confiável adaptado para ser autoadministrado em homens com sintomas de STUI, especialmente aqueles com câncer ^( 17 )^. O efeito significativo das DS relacionado aos sintomas de STUI destaca a necessidade de os profissionais de saúde avaliarem a gravidade desse impacto e personalizar as abordagens terapêuticas ^( 28 )^. Nesse contexto, são propostas medidas PROMs para pessoas com DS relacionadas a STUI para melhorar tanto a gravidade quanto a avaliação pós-terapia ^( 5 )^.

Embora seja reconhecida a importância da adaptação cultural dos questionários, a falta de adequação nos procedimentos de adaptação segue sendo uma preocupação para os investigadores. Uma adaptação correta requer a consideração cuidadosa de diversos fatores, como elementos culturais, idiomáticos, linguísticos e contextuais relacionados à tradução do instrumento, o que é essencial ^( 16 )^.

Na versão original do ICIQ-MLUTSsex ^( 8 )^, foi utilizado o modelo de validação triangular delineado na Teoria Clássica dos Testes (validade de conteúdo, critério e construto) ^( 29 )^. Para estabelecer a capacidade do questionário em mensurar diversas DS, os criadores avaliaram sua validade de construto correlacionando os escores dos itens com os sintomas relatados pelos participantes, ainda que sem examinar sua estrutura interna ^( 8 )^.

A atual teoria de medição enfatiza que qualquer instrumento concebido para medir um construto deve ter uma estrutura interna que reflita a realidade empírica desse construto. Isto é fundamental para interpretar os escores obtidos na aplicação do instrumento ^( 30 )^. Consequentemente, as evidências de validade da estrutura interna indicam o grau em que a teoria do construto apoia e elucida os resultados de um teste. As evidências podem ser estabelecidas através de análises de AFE e AFC ^( 31 )^.

O ICIQ-MLUTSsex-Versão Brasileira foi avaliado em termos de evidências de sua estrutura interna com o auxílio da psicometria moderna. Essa abordagem é considerada padrão-ouro no desenvolvimento, validação e utilização de testes, garantindo que os testes sejam equitativos, confiáveis e válidos ^( 17 )^. Para evidências de avaliação de validade, utilizou-se matriz de correlação policórica devido à natureza ordinal dos dados ^( 24 )^. Esta matriz apresentou resultados favoráveis, corroborados pelos índices de adequação satisfatórios. O ICIQ-MLUTSsex-Versão Brasileira foi estruturado como unidimensional, com cargas fatoriais elevadas e comunalidades adequadas ^( 18 )^. Os itens 2a, 3a e 5a apresentaram cargas fatoriais próximas de 1 e alta comunalidade, indicando que pode se tratar de aspectos de mensuração do construto muito semelhantes. Isso sugere que esses itens devem ser o ponto central de pesquisas em estudos futuros com amostras diversas para confirmar seu comportamento. Por meio da AFE, identificamos uma proporção de variância comum satisfatória que representou 78% da variância explicada ^( 32 )^, o que corrobora sua consistência e robustez para mensuração do construto DS em homens com STUI.

Quanto às cargas fatoriais, os valores foram aceitáveis dependendo do tamanho da amostra (0,50 para amostras com 120 a 150 participantes) ^( 18 )^. Os valores de comunalidade correspondentes aos itens foram considerados indicadores da variabilidade explicada pelos fatores, proporcionando assim uma avaliação da qualidade do modelo com um único fator ^( 18 )^. De fato, os índices UniCO, VCE e MIREAL confirmaram a unidimensionalidade do instrumento, além disso, a replicabilidade foi confirmada através do *Construct Reliability – Index G H*. A AFC corroborou o modelo unidimensional do ICIQ-MLUTSsex-Versão Brasileira com quatro itens que apresentaram cargas fatoriais significativas. Isso mostra que a estrutura interna do módulo de DS representa a construção. Embora o índice de ajuste RMSEA tenha sido superior a 0,05, essa discrepância foi atribuída ao tamanho insuficiente da amostra deste estudo para estimar esse índice com precisão ^( 18 , 25 )^. Esses resultados são consistentes com a teoria que sustenta os itens do instrumento original ^( 8 )^, baseado nos quatro principais sintomas que representam DS associados aos sintomas de STUI ^( 6 )^.

O grau de validade varia nos instrumentos de medida, uma vez que as propriedades psicométricas não são estáticas e dependem da interação entre a população com a qual o instrumento é utilizado e as circunstâncias em que é utilizado ^( 33 )^. Consequentemente, a análise da estrutura interna sugeriu que as quatro perguntas feitas no estudo original ^( 8 )^ foram eficazes na medição e explicação destes sintomas em homens com STUI.

Quanto à confiabilidade, o α de Cronbach foi superior ao da amostra original do estudo ^( 8 )^ e, quando combinado com o ω de McDonald ^( 17 , 27 )^, confirmou a confiabilidade do ICIQ-MLUTSsex-Versão Brasileira. Isso significa que os itens possuem uma inter-relação uniforme para avaliar o construto DS em homens com STUI e que os escores obtidos pelos respondentes não variam de forma significativa ao longo do tempo.

As evidências de validade na relação com variáveis externas verificaram ligação direta entre sintomas prostáticos e DS. Esses achados são consistentes com a literatura existente ^( 2 - 3 )^, apoiando a noção de que os sintomas da próstata predizem DS.

Apesar dos resultados promissores, uma das limitações deste estudo reside no fato de as etapas de pré-teste e validação empírica terem sido realizadas apenas em um ambulatório de Urologia de um hospital especializado em Oncologia, onde mais da metade da amostra era composta por homens com câncer. Consequentemente, estudos futuros deverão ampliar suas amostras para verificar a validade dos achados em diferentes regiões do Brasil e entre diferentes populações no campo das doenças urológicas. Outra limitação foi que o estudo original não permitiu comparação direta com a versão brasileira do instrumento, principalmente porque as variáveis utilizadas para validar a relação com variáveis externas foram derivadas de exames de urofluxometria ^( 8 )^. A ausência de dados de urofluxometria nos prontuários impediu a replicação da análise de evidências do estudo original, que envolveu a comparação dos achados com diários de frequência-volume e resultados da análise de urofluxometria ^( 8 )^.

O ICIQ-MLUTSsex-Versão Brasileira traz importantes contribuições teóricas ao processo de adaptação transcultural desse instrumento. Essa versão destaca-se por ter sido submetida a análises psicométricas robustas, que confirmaram a presença de uma estrutura interna que fundamenta a interpretação dos seus itens. Em contraste com esta abordagem abrangente, as adaptações para a África do Sul, Canadá, Estados Unidos, Coreia, México e Eslováquia fizeram parte de um estudo multicêntrico com uma amostra modesta de 30 participantes. Este estudo focou principalmente nos aspectos linguísticos para garantir que os itens fossem de fácil compreensão ^( 9 )^, sem se aprofundar em quaisquer verificações de outros tipos de evidências de validade necessárias para medir com precisão o construto.

A utilização deste instrumento abrirá novos horizontes interpretativos sobre as DS em pessoas com STUI no Brasil. Seus resultados poderão subsidiar as ações de cuidado dos enfermeiros nos diferentes ambientes de saúde, bem como direcionar medidas para desenhar estratégias mais eficazes para reduzir o efeito das DS na qualidade de vida dos homens. Outrossim, este trabalho de pesquisa apresenta técnicas sólidas, considerando as recomendações psicométricas contemporâneas.

## Conclusão

Este estudo estabelece que o ICIQ-MLUTSsex-Versão Brasileira para homens que sofrem de STUI (especialmente pacientes com câncer) apresenta evidências sólidas de validade e confiabilidade. Isto representa um avanço significativo porque fornece um novo caminho de pesquisa para a avaliação inicial de DS em brasileiros com STUI, uma vez que preenche a lacuna deixada pela ausência de outras ferramentas validadas que relacionem STUI com DS. Espera-se que esta medida PROMs seja extremamente útil em futuras pesquisas e práticas clínicas.

# Translation: Validez y fiabilidad de la versión brasileña del módulo Problemas Sexuales Masculinos Asociados a Síntomas del Tracto Urinario Inferior del Cuestionario de Consulta internacional sobre Incontinencia*

**DOI:** 10.1590/1518-8345.7481.4544

**Authors:** André Carlos Santos Ferreira, Dayane Abreu Ribeiro, José Wicto Pereira Borges, M. Graça Pereira, Kátia Santana Freitas, Luciana Regina Ferreira da Mata

**Affiliations:** 1 Universidade Federal de Minas Gerais, Escola de Enfermagem, Belo Horizonte, MG, Brasil; 2 Becario de la Coordenação de Aperfeiçoamento de Pessoal de Nível Superior (CAPES), Brasil; 3 Becaria de la Fundação de Amparo à Pesquisa do Estado de Minas Gerais (FAPEMIG), Brasil; 4 Universidade Federal do Piauí, Escola de Enfermagem, Floriano, PI, Brasil; 5 Universidade do Minho, Escola de Psicologia, Braga, Minho, Portugal; 6 Universidade Estadual Feira de Santana, Departamento de Saúde, Feira de Santana, BA, Brasil

**Keywords:** Salud del Hombre, Disfunciones Sexuales Fisiológicas, Estudio de Validación, Síntomas del Sistema Urinario Inferior, Medición de Resultados Informados por el Paciente, Encuestas y Cuestionarios

## Abstract

describir el proceso de adaptación cultural del módulo de Problemas Sexuales Masculinos Asociados a Síntomas del Tracto Urinario Inferior del Cuestionario de Consulta internacional sobre Incontinencia, al igual que analizar la validez y fiabilidad de su versión brasileña en hombres que padecen síntomas urinarios.

este estudio transversal se realizó con 138 hombres que sufren síntomas urinarios sometidos a control en la clínica ambulatoria de Urología de un hospital especializado en Oncología. La evidencia de validez de la estructura interna se evaluó por medio de los cuatro ítems del cuestionario, a través de análisis factorial tanto exploratorio como confirmatorio. Además, la fiabilidad se analizó empleando los coeficientes α de Cronbach y ω de McDonald. La evidencia de validez en la relación con variables externas se evaluó utilizando correlación de Spearman con la Puntuación de síntomas prostáticos (*International Prostate Symptom Score*) y el Cociente sexual masculino (*Male Sexual Quotient*).

se detectó significativa evidencia de validez de contenido, proceso de respuesta y un único factor que explicó el 78% de la varianza, con cargas factoriales que oscilaron entre 0,54 y 0,97. Los coeficientes α de Cronbach y ω de McDonald alcanzaron valores cercanos a 0,95, lo que indica un nivel satisfactorio de fiabilidad. Se encontró una correlación positiva moderada entre el cuestionario utilizado y la puntuación *International Prostate Symptom Score*(r=0,67), al igual que una fuerte correlación negativa con el cociente *Male Sexual Quotient*(r=-0,84), lo que sustenta buena evidencia de validez en la relación con variables externas.

la versión brasileña del cuestionario presentó sólida evidencia de validez y fiabilidad para evaluar disfunciones sexuales en la población de estudio.

## Destacados

**(1)** ICIQ-MUTSsex es un cuestionario de evaluación válido y fiable. **(2)** ICIQ-MUTSsex puede utilizarse en el ámbito de la investigación y en la práctica clínica. **(3)** El cuestionario evalúa disfunciones sexuales en brasileños que sufren síntomas urinarios.

## Introducción

Los síntomas del tracto urinario inferior (STUI) representan un término general que abarca variados problemas urinarios, categorizados en síntomas de almacenamiento o irritantes, de vaciado u obstructivos y posteriores al vaciado ^( 1 )^. La prevalencia de los STUI aumenta con la edad y afecta al 69% de los hombres de al menos 40 años de edad en Brasil ^( 2 )^. Los síntomas están asociados a dificultades sexuales y trastornos en la próstata, afectando así la calidad de vida (CdV) masculina ^( 3 )^.

Para los hombres, la identidad sexual y la función eréctil son primordiales para mantener su vida sexual. Las disfunciones sexuales (DS) se caracterizan por alteraciones en al menos una de las fases del ciclo de respuesta sexual, que incluye las etapas de deseo, excitación y orgasmo ^( 4 )^. La prevalencia de las DS está directamente relacionada con la incidencia de los STUI, particularmente en hombres de más de 40 años ^( 2 )^. Por ejemplo: en un metaanálisis de 24 estudios se reportó que los síntomas STUI más graves están asociados a un mayor riesgo de desarrollar diversas DS (es decir, síntomas STUI más graves se correlacionaron con niveles más bajos de función eréctil y satisfacción sexual) ^( 3 )^.

Desde este punto de vista, considerando la relación entre síntomas STUI y las DS y el efecto de estas enfermedades sobre la CdV masculina, queda en evidencia la necesidad de que los profesionales de la salud evalúen las DS en hombres con problemas relacionados con síntomas STUI ^( 3 )^. Las Medidas de resultado reportadas por los pacientes (*Patient-Reported Outcome Measures*, PROM) ofrecen una alternativa para evaluar información subjetiva con objetividad. Estas herramientas sustentan la toma de decisiones clínicas y permiten comprender mejor el efecto de estas enfermedades sobre la CdV de una persona ^( 5 )^. En una búsqueda en la literatura para identificar medidas PROM enfocadas en el constructo de las DS se encontraron 23 instrumentos, aunque solo dos de ellos asociaban las DS con los STUI ^( 6 - 7 )^. En este contexto, el módulo de Problemas sexuales masculinos asociados a síntomas del tracto urinario inferior del cuestionario de Consulta internacional sobre incontinencia (*International Consultation on Incontinence Questionnaire Male Sexual Matters Associated with Lower Urinary Tract Symptoms Module*, ICIQ-MLUTSsex) se destaca ^( 6 , 8 )^ como un instrumento altamente recomendado (Nivel A) ^( 1 )^. Originalmente desarrollado en el año 1996 por académicos británicos ^( 8 )^, el nombre inicial de ICIQ-MLUTSsex era *International Continence Society - Sexual Function Questionnaire*(ICSsex). En 1999 e iniciado por la Sociedad Internacional de Continencia (*International Continence Society*, ICS), el proyecto del cuestionario de Consulta internacional sobre incontinencia (*International Consultation on Incontinence Questionnaire*, ICIQ) tuvo como objetivo desarrollar y validar medidas PROM para pacientes que padecían disfunción del tracto urinario inferior, síntomas vaginales y disfunción del intestino inferior. Al lanzarse el proyecto, se cambió el nombre del cuestionario ICSsex por ICIQ-MLUTSsex ^( 1 )^.

ICIQ-MLUTSsex es un cuestionario breve, sencillo y autoadministrado que se desarrolló para evaluar diversas DS en hombres con STUI. Aunque sus propiedades psicométricas solo se han reportado en su versión original ^( 8 )^, se lo ha adaptado transculturalmente en Canadá, Sudáfrica, Estados Unidos, Corea del Sur, Eslovaquia y México ^( 9 )^, además de ser objeto de diversos estudios clínicos ^( 10 - 15 )^. En Brasil no se conoce ningún instrumento para evaluar DS en hombres con STUI ni evaluar el efecto de las DS sobre la CdV. Por lo tanto, el propósito de este estudio fue describir el proceso de adaptación cultural de ICIQ-MLUTSsex y analizar la validez y fiabilidad de su versión brasileña en hombres con síntomas del tracto urinario inferior.

## Método

### Diseño del estudio

Con la debida autorización del grupo ICIQ, este estudio se realizó en dos etapas. En la primera, el estudio metodológico siguió las recomendaciones para realizar adaptaciones transculturales (traducción, retrotraducción, evaluación a cargo de un comité de expertos y pasos para pruebas previas) ^( 16 )^. La segunda etapa consistió en un estudio observacional y transversal enfocado en el análisis de las propiedades psicométricas. Se verificó evidencia de validez de contenido, proceso de respuesta, estructura interna y relación con variables externas ^( 17 )^.

### Sede del estudio

El estudio se desarrolló con pacientes sometidos a control en la clínica ambulatoria de Urología de un hospital especializado en Oncología de Belo Horizonte (estado de Minas Gerais, Brasil).

### Período

Los datos se recolectaron entre enero y abril de 2024.

### Población, criterios de elegibilidad y muestra

La población del estudio estuvo compuesta por hombres de 18 años o más que manifestaban padecer al menos un STUI ^( 1 )^, sometidos a control en la clínica ambulatoria de Urología de un hospital especializado en Oncología. Se excluyó a hombres con sondas urinarias permanentes.

En estudios psicométricos, el tamaño de la muestra se estima en función de la cantidad de ítems, lo que evidencia relaciones de 20:1 ^( 18 )^. Se necesitó una muestra mínima de 80 participantes porque el instrumento tiene cuatro ítems. Se reclutó una muestra no probabilística y secuencial conformada por 138 hombres. En consecuencia, la cantidad de participantes fue adecuada sobre la base de las recomendaciones indicadas en la literatura ^( 18 )^.

### Instrumento original

ICIQ-MLUTSsex forma parte de un cuestionario más extenso (*ICS–“BPH”Study*) ^( 8 )^, cuya estructura cuenta con tres subescalas que evalúan síntomas urinarios (22 ítems), su efecto sobre la CdV (siete ítems) y las DS (cuatro ítems). En este estudio se consideraron los cuatro ítems de ICSmale que cubren el funcionamiento sexual. El cuestionario ICIQ-MLUTSsex incluye cuatro ítems que evalúan la erección, la eyaculación, el dolor durante la eyaculación y el efecto de los STUI sobre la CdV. Cada ítem se responde mediante una escala Likert con cuatro opciones que varían de cero (sin cambios) a tres (síntomas graves). La puntuación total varía de cero a 12 puntos, donde valores más altos denotan problemas sexuales más severos. Además, el cuestionario incluye escalas de molestia para cada ítem (de cero a 10), donde cero indica “ningún problema” y 10 representa “un problema grave”. Las escalas de molestia no se integran a la puntuación final, pero indican el efecto de cada síntoma sobre el paciente ^( 6 , 8 )^.

### Adaptación transcultural

Como primera medida, se solicitó aprobación para usar y adaptar ICIQ-MLUTSsex al grupo ICIQ. En el proceso de adaptación transcultural se siguió un método estandarizado ^( 16 )^, que se considera en el protocolo de traducciones de ICIQ. Los encargados de traducir ICIQ-MLUTSsex al portugués de Brasil fueron dos traductores brasileños con fluidez en inglés, generando así dos versiones independientes (T1 y T2). Posteriormente, un urólogo bilingüe combinó ambas versiones en una (T1+T2). Otros dos traductores brasileños con perfecto dominio del inglés retrotradujeron esta versión a ese idioma, con lo que se generaron dos versiones retrotraducidas independientes (RT1 y RT2). El grupo ICIQ revisó las versiones retrotraducidas y sugirió tres cambios: 1) refrasear los ítems 2b, 3b, 4b y 5b; 2) reemplazar la traducción al portugués correspondiente a “*hampered*” (“obstaculizado”) y “*affected*” (“afectado”) en el ítem 5a por una palabra que reflejara con mayor exactitud el efecto negativo de los síntomas urinarios sobre la vida sexual; y 3) proponer que se retire el término “*ativa*” (“activa”) del ítem 5c. Sin embargo, quien introdujo este término durante la síntesis de las traducciones en la segunda etapa fue un traductor con título de médico y Doctorado en Urología. En consecuencia, decidimos conservarlo para que los participantes comprendieran mejor el contenido. Después de estas modificaciones, el grupo ICIQ aprobó la versión en portugués de Brasil.

Posteriormente, un panel compuesto por 20 expertos brasileños (un especialista en Lingüística, tres urólogos, ocho especialistas en Psicometría, seis enfermeros y dos sexólogos) evaluó la validez del contenido en términos de claridad, relevancia teórica y facilidad de aplicación práctica de los ítems examinados. Se calculó la razón de validez de contenido (CVR) para cada ítem; se consideraron aceptables valores de CVR iguales o superiores a 0,474 ^( 19 )^. Se realizó una prueba previa con 39 hombres que sufrían STUI ^( 16 )^. Durante las entrevistas, se preguntó a los participantes acerca de la claridad y relevancia teórica de los ítems, además de preguntarles si las opciones de respuesta eran claras y fáciles de entender. En esta etapa se realizaron entrevistas cognitivas y semiestructuradas para evaluar la versión pre-final. Todas las entrevistas fueron individuales y estuvieron a cargo de un solo entrevistador debidamente capacitado. En el último paso, se envió la versión de ICIQ-MLUTSsex traducida al portugués de Brasil a los desarrolladores originales del instrumento y se evaluaron sus propiedades psicométricas.

### Recolección de datos

Se recopilaron datos sobre factores sociodemográficos y clínicos (edad, estado civil, nivel de estudios, diagnóstico médico, síntomas STUI y comorbilidades). Los STUI se evaluaron sobre la base de síntomas de almacenamiento (p. ej.: polaquiuria, nocturia, urgencia e incontinencia urinaria), síntomas de micción (p. ej.: chorro de orina lento, chorro de orina intermitente, duda al orinar, esfuerzo al orinar y goteo final) y síntomas posteriores a la micción (p. ej.: goteo después de orinar y sensación de vaciado incompleto) ^( 1 )^. Cada participante respondió los cuestionarios individualmente, en un lugar privado y sin ningún límite de tiempo.

La puntuación *International Prostate Symptom Score* incluye siete preguntas enfocadas en síntomas urinarios comúnmente asociados a hiperplasias prostáticas benignas, además de una pregunta aparte sobre la CdV en relación a estos síntomas. Cada pregunta se responde mediante una escala Likert de 0 (Nunca) a 5 (Casi siempre o Al menos cinco veces). La puntuación acumulada de IPSS es la suma de los siete puntajes individuales y varía de cero a 35. Cuanto más alta es la puntuación, más grave es el síntoma ^( 20 - 21 )^.

El cociente *Male Sexual Quotient* consiste en 10 preguntas autoinformadas que evalúan aspectos como deseo e interés sexual (pregunta 1), confianza en uno mismo (pregunta 2), calidad de la erección (preguntas 5 a 7), control de la eyaculación (pregunta 8), capacidad para llegar al orgasmo (pregunta 9) y nivel de satisfacción, tanto para el hombre (preguntas 3, 4 y 10) como para la pareja (preguntas 3 y 10). Los ítems se responden con una escala Likert de cinco puntos, donde 0 indica “Nunca” y 5 denota “Siempre”. Puntuaciones más elevadas indican mayor satisfacción sexual. La puntuación total se calcula sumando los valores obtenidos en las preguntas 1 a 10 y multiplicando por dos; de esta manera, se genera una escala de 0 a 100 en la que cuanto más alta es la puntuación, mejor es el desempeño sexual ^( 22 - 23 )^.

### Procesamiento y análisis de los datos

La evidencia de validez de la estructura interna se evaluó por medio de un análisis factorial exploratorio, seguido por otro confirmatorio. La dimensionalidad se evaluó a través de Congruencia unidimensional (*Unidimensional COngruence*, UniCO) ≥0,95, Varianza Común Explicada (VCE) ≥0,85 y *Mean of Item Residual Absolute Loading* (MIREAL) ≤0,30 ^( 24 )^. La calidad y efectividad de las estimaciones factoriales se midieron con *Factor Determinacy Index*(FDI) >0,80, Relación de sensibilidad (RS) >0,2 e *Expected Percentage of True Differences*(EPTD) >90% ^( 24 )^. La evaluación con respecto a mantener o excluir ítems se basó en los siguientes criterios: saturación de cargas factoriales; carga factorial ≥0,50; evaluación de ítems con cargas transversales; y comunalidades (h^2^) ≥0,40; al igual que en la relevancia práctica y conceptual de cada ítem de acuerdo con el constructo a medir. Se midieron los índices de ajuste del modelo, como *Root Mean Square Error of Approximation* (RMSEA) <0,05, contenido de ajuste >0,95 e Índice Tucker-Lewis >0,95 ^( 25 )^. La correlación de Spearman entre las puntuaciones de ICIQ-MLUTSsex-Versión brasileña e IPSS y MSQ definió evidencia de validez en la relación con variables externas. Coeficientes de correlación de 0,40 a 0,69 indicaron correlaciones moderadas, mientras que valores de al menos 0,70 señalaron correlaciones fuertes ^( 26 )^. Se formularon las siguientes dos hipótesis: a) Hay una correlación positiva entre la puntuación total obtenida en ICIQ-MLUTSsex-Versión brasileña y la puntuación IPSS; y b) Hay una correlación negativa entre la puntuación total obtenida en ICIQ-MLUTSsex-Versión brasileña y la puntuación MSQ. La fiabilidad se evaluó utilizando los coeficientes ω de McDonald y α de Cronbach, considerando 0,70 como valor satisfactorio ^( 27 )^. Se emplearon los programas de software Factor (versión 10.10.03) y Mplus (versión 7.4) para el análisis estadístico.

### Aspectos éticos

El proyecto de investigación fue aprobado por el Comité de Ética en Investigación de una institución de enseñanza (N.º 5.287.948) conforme a las recomendaciones estipuladas en la Resolución 466/2012 del Concejo de Salud de Brasil. Todos los participantes firmaron un formulario de Consentimiento informado.

## Resultados

### Evidencia de validez de contenido

Un comité compuesto por 20 expertos evaluó la validez de contenido de ICIQ-MLUTSsex-Versión brasileña durante el proceso de adaptación transcultural y a través de entrevistas cognitivas con hombres que padecen síntomas STUI. El comité de expertos considero la validez de contenido de ICIQ-MLUTSsex-Versión brasileña como muy buena. Sugirieron excluir la traducción de ‘*discomfort*’ (‘molestia’) al portugués del ítem 3a debido a su CVR de 0,473. Se realizaron entrevistas cognitivas con 39 hombres que padecían síntomas STUI, de 33 a 92 años de edad (mediana=65; DE=11,07) y con distintos niveles de estudios, razas y estados civiles. El análisis de las entrevistas cognitivas reveló que los ítems 2ª (*Você tem ereções?* [¿Tiene erecciones?]), 3ª (*Você tem ejaculação de sêmen* [¿Eyacula semen?]) y 4ª (*Você sente dor durante a ejaculação de sêmen* [¿Siente dolor al eyacular?]) no eran claros; eso llevó a incluir los términos ‘*pênis duro*’ (‘pene duro’) en el ítem 2a, ‘*você goza*’ (‘eyacula’) en el ítem 3a y ‘*ao gozar*’ (‘cuando eyacula’) en el ítem 4a como sinónimos de las palabras confusas. Se envió la versión final de ICIQ-MLUTSsex-Versión brasileña a los desarrolladores originales del instrumento (quienes la aprobaron) y se evaluaron sus propiedades psicométricas.

### Características de la muestra

Un total de 138 hombres de 27 a 80 años de edad participaron del estudio, en su mayoría (70.3%) personas diagnosticadas con cáncer. En la Tabla 1 se indican las características sociodemográficas y clínicas de los pacientes.

**Tabla 1 -:** Características sociodemográficas y clínicas de los participantes (n = 138). Belo Horizonte, MG, Brasil, 2023

**Características**	
Edad (años), mediana (mínimo-máximo)	61 (27-80)
Estado civil, n* (% ^†^ )	
Con pareja	110 (79,7)
Nivel de estudios, n* (% ^†^ )	
Escuela Primaria	83 (60,2)
Escuela Secundaria	47 (34,1)
Educación Superior	08 (5,7)
Diagnóstico médico, n* (% ^†^ )	
Cáncer de próstata	67 (48,6)
Hiperplasia prostática benigna	41 (29,7)
Cáncer de vejiga	12 (8,7)
Cáncer de pene	10 (7,2)
Cáncer de testículo	03 (2,2)
Cáncer de riñón	03 (2,2)
Cáncer de hígado	01 (0,7)
Cáncer de cuello	01 (0,7)
Comorbilidades, n* (% ^†^ )	
Hipertensión	26 (18,9)
Diabetes	27 (19,5)
Hipertensión y diabetes	31 (22,4)
No corresponde	54 (39,2)
Síntomas del tracto urinario inferior, n* (% ^†^ )	
Almacenamiento + micción + posmicción	80 (58,1)
Almacenamiento + micción	47 (34,1)
Almacenamiento	6 (4,3)
Almacenamiento + posmicción	2 (1,4)
Micción	2 (1,4)
Micción + posmicción	1 (0,7)

*n = Tamaño de la muestra; ^†^% = Porcentaje

### Evidencia de validez de la estructura interna

En el proceso de validación se demostró que el análisis factorial para la matriz de datos de ICIQ-MLUTSsex-Versión brasileña fue apropiado (Kaiser-Meyer-Olkin=0,81; prueba de esfericidad de Bartlett, *p*<0,05; determinante de la matriz de correlación=0,02). Tal como se presenta en la Tabla 2, el análisis sugirió que ICIQ-MLUTSsex-Versión brasileña es un instrumento unidimensional. El modelo con cuatro ítems se consideró adecuado para evaluar indicadores de unidimensionalidad; además, los índices UniCO, VCE y MIREAL corroboraron la unidimensionalidad de ICIQ-MLUTSsex-Versión brasileña. El análisis reveló un alto porcentaje de varianza explicada (78%) para la dimensión 1. Considerando cargas factoriales y comunalidades, no se excluyó ningún ítem, confirmando así la idoneidad del modelo del cuestionario ICIQ-MLUTSsex-Versión brasileña.

**Tabla 2 -:** Análisis factoriales exploratorio y confirmatorio para evaluar la estructura dimensional de ICIQ-MLUTSsex-Versión brasileña. Belo Horizonte, MG, Brasil, 2023

Ítems		**AFE** *		**AFC** ^†^	
		λ ^‡^	h ^2§^	λ ^‡^	***δ*** ^||^
2a	*Você tem ereções (pênis duro)?*	0,97	0,94	0,97	0,05
3a	*Você tem ejaculação de sêmen (você goza)?*	0,92	0,85	0,95	0,08
4a	*Você sente dor durante a ejaculação de sêmen (ao gozar)?*	0,54	0,40	0,76	0,41
5a	*O quanto você sente que sua vida sexual foi prejudicada por seus sintomas urinários?*	0,91	0,83	0,94	0,09
Determinante de la matriz de correlación		0,02			
Prueba de esfericidad de Bartlett		*p* <0,05			
Índice Kaiser-Meyer-Olkin		0,81			
χ ^2^ ¶				7,19 ( *p* =0,02)	
RMSEA**				0,13	
IAC ^††^				0,99	
ITL ^‡‡^				0,99	
Unidimensionalidad					
UniCO ^§§^		0,99			
VCE ^||||^		0,94			
MIREAL ^¶¶^		0,18			

*AFE = Análisis Factorial Exploratorio; ^†^AFC = Análisis Factorial Confirmatorio; ^‡^λ = Cargas factoriales; ^§^h^2^ = Comunalidades; ^||^ ***δ*** = Varianza residual; ^¶^χ^2^ = Chi cuadrado; **RMSEA =*Root Mean Square Error of Approximation*; ^††^IAC = Índice de Ajuste Comparativo; ^‡‡^ITL = Índice Tucker-Lewis; ^§§^UniCO = Congruencia unidimensional; ^||||^VCE = Varianza Común Explicada; ^¶¶^MIREAL = *Mean of Item Residual Absolute Loading*

### Evidencia de validez en la relación con variables externas

Se registró una correlación positiva moderada entre todo el instrumento ICIQ-MLUTSsex-Versión brasileña e IPSS (r=0,67; p<0,05) y una fuerte correlación negativa entre la totalidad de ICIQ-MLUTSsex-Versión brasileña y MSQ (r=-0,84; p<0,05); eso indica que hubo una relación directa entre las variables, confirmando así las hipótesis establecidas.

### Fiabilidad

La fiabilidad se evaluó utilizando los coeficientes ω de McDonald y α de Cronbach, lo que arrojó resultados de 0,95 y excelente replicabilidad, tal como se confirmó con *Construct Reliability – Index GH*. Los índices FDI, EPTD y RS también indicaron buena estimación de factores (Tabla 3).

En vista de los resultados obtenidos, en la Figura 1 se muestra la versión final de ICIQ-MLUTSsex-Versión brasileña.

**Tabla 3 -:** Índices de fiabilidad, calidad y efectividad correspondientes a ICIQ-MLUTSsex-Versión brasileña. Belo Horizonte, MG, Brasil, 2023

**Técnicas utilizadas**	Índices	**Resultados**
Fiabilidad	Alfa de Cronbach	0,95
	Omega de McDonald	0,95
Calidad y eficiencia	*Construct Reliability - Index* G H	0,81
	FDI*	0,98
	EPTD ^†^	100,0% ^‡^
	RS ^§^	41,19

*FDI = *Factor Determinacy Index*; ^†^EPTD = *Expected Percentage of True Differences*; ^‡^% = Porcentaje; ^§^RS = Relación de sensibilidad

**Figura 1 -:** ICIQ-MLUTSsex-Versión brasileña. Belo Horizonte, MG, Brasil, 2023

## Discusión

El objetivo de este estudio fue adaptar culturalmente ICIQ-MLUTSsex-Versión brasileña y analizar su validez y fiabilidad en hombres que padecen síntomas STUI, en su mayoría (70,3%) pacientes con cáncer. ICIQ-MLUTSsex-Versión brasileña es el primer cuestionario adaptado al portugués de Brasil para evaluar DS en hombres con síntomas STUI y el efecto de las DS sobre su CdV ^( 6 )^. En el proceso de adaptación transcultural al portugués de Brasil, el comité de expertos solicitó retirar el término en portugués correspondiente a “*discomfort*” del ítem 3a. En la prueba previa, sugirieron añadir los términos “*pênis duro*”, “*você goza*” y “*ao gozar*” a los ítems 2a, 3a y 4a, respectivamente. Sin embargo, a diferencia del estudio de validación de ICIQ-MLUTSsex al coreano, no se modificaron las opciones de respuesta para el ítem 2a ^( 9 )^. Los cambios hechos en la adaptación para Brasil se reflejaron en la evaluación positiva sobre la claridad de las preguntas, en la relevancia teórica de los ítems y en definir si las opciones de respuesta de ICIQ-MLUTSsex-Versión brasileña eran claras y fáciles de entender durante las entrevistas cognitivas.

En el proceso de adaptación transcultural se respetaron pautas específicas ^( 16 )^; eso derivó en un instrumento válido y fiable adaptado para ser autoadministrado en hombres con síntomas STUI, especialmente aquellos con cáncer ^( 17 )^. El significativo efecto de las DS relacionadas con síntomas STUI destaca la necesidad de que los profesionales de la salud evalúen la gravedad de este impacto y personalicen enfoques terapéuticos en consonancia ^( 28 )^. En este contexto, se proponen medidas PROM para personas con DS relacionadas con síntomas STUI para mejorar tanto la evaluación de la gravedad como la posterior a la terapia ^( 5 )^.

Aunque se reconoce la importancia de adaptar cuestionarios culturalmente, la falta de idoneidad en los procedimientos de adaptación sigue preocupando a los investigadores. Una adaptación correcta requiere considerar cuidadosamente varios factores, como ser elementos culturales, idiomáticos, lingüísticos y contextuales relacionados con la traducción del instrumento, lo que es primordial ^( 16 )^.

En la versión original de ICIQ-MLUTSsex ^( 8 )^ se empleó el modelo de validación triangular delineado en la Teoría Clásica de los *Tests* (contenido, criterio y validez de constructo) ^( 29 )^. A fin de establecer la capacidad del cuestionario para medir diversas DS, los creadores evaluaron su validez de constructo correlacionando las puntuaciones de los ítems con los síntomas informados por los participantes, aunque sin examinar su estructura interna ^( 8 )^.

La teoría de medición actual destaca que cualquier instrumento diseñado para medir un constructo debe poseer una estructura interna que refleje la realidad empírica de ese constructo. Eso es crucial para interpretar las puntuaciones obtenidas al aplicar el instrumento ^( 30 )^. En consecuencia, la evidencia de validez de la estructura interna señala el grado en el cual la teoría de constructo sustenta y elucida los resultados de una prueba. La evidencia se puede establecer a través de análisis AFE y AFC ^( 31 )^.

ICIQ-MLUTSsex-Versión brasileña se evaluó en términos de evidencia de su estructura interna utilizando psicometría moderna. Se considera que este enfoque es el estándar de oro en el desarrollo, la validación y la utilización de pruebas, garantizando así que los *tests* sean equitativos, fiables y válidos ^( 17 )^. Para la evidencia de evaluación de la validez se empleó una matriz de correlación policórica debido a la naturaleza ordinal de los datos ^( 24 )^. Esta matriz arrojó resultados favorables, tal como lo sustentan los índices de idoneidad satisfactorios. ICIQ-MLUTSsex-Versión brasileña se estructuró como unidimensional, con cargas factoriales elevadas y comunalidades adecuadas ^( 18 )^. Los ítems 2a, 3a y 5a presentaron cargas factoriales cercanas a 1 y alta comunalidad, indicando así que pueden ser aspectos de medición del constructo muy similares. Eso sugiere que estos ítems deberían ser el punto central de investigación en próximos estudios con muestras diversas para confirmar su comportamiento. A través del AFE, identificamos una proporción de varianza común satisfactoria que representó el 78% de la varianza explicada ^( 32 )^, lo que sustenta su consistencia y solidez para medir el constructo de DS en hombres con síntomas STUI.

En lo que respecta a las cargas factoriales, los valores fueron aceptables en función del tamaño de la muestra (0,50 para muestras con 120 a 150 participantes) ^( 18 )^. Los valores de comunalidad correspondientes a los ítems se consideraron indicadores de la variabilidad explicada por los factores, proporcionando de esta manera una evaluación de la calidad del modelo con un solo factor ^( 18 )^. Es más, los índices UniCO, VCE y MIREAL confirmaron la unidimensionalidad del instrumento, además, la replicabilidad se confirmó a través de *Construct Reliability – Index GH*. El AFC corroboró el modelo unidimensional de ICIQ-MLUTSsex-Versión brasileña con cuatro ítems que tuvieron cargas factoriales significativas; eso demuestra que la estructura interna del módulo para las DS representa el constructo. Si bien el índice de ajuste RMSEA fue superior a 0,05, esta discrepancia se atribuyó al tamaño insuficiente de la muestra de este estudio para estimar este índice con exactitud ^( 18 , 25 )^. Estos resultados se condicen con la teoría que sustenta a los ítems del instrumento original ^( 8 )^, basada en los cuatro síntomas principales que representan a las DS asociadas con síntomas STUI ^( 6 )^.

El grado de validez varía en los instrumentos de medición, ya que las propiedades psicométricas no son estáticas y dependen de la interacción entre la población con la que se utiliza el instrumento y las circunstancias en las que se emplea ^( 33 )^. En consecuencia, el análisis de la estructura interna sugirió que las cuatro preguntas formuladas en el estudio original ^( 8 )^ fueron efectivas para medir y explicar estos síntomas en hombres con STUI.

En cuanto a la fiabilidad, el α de Cronbach fue superior al de la muestra del estudio original ^( 8 )^ y, al combinarlo con el ω de McDonald ^( 17 , 27 )^, confirmó la fiabilidad de ICIQ-MLUTSsex-Versión brasileña. Eso significa que los ítems poseen una interrelación uniforme para evaluar el constructo de DS en hombres con síntomas STUI y que las puntuaciones obtenidas por los encuestados no cambian tanto con el paso del tiempo.

La evidencia de validez en la relación con variables externas verificó una conexión directa entre los síntomas prostáticos y las DS. Estos hallazgos se condicen con la literatura existente ^( 2 - 3 )^, lo que respalda la noción de que los síntomas prostáticos predicen las DS.

Pese a los resultados prometedores, una de las limitaciones de este estudio radica en el hecho de que las etapas de prueba previa y validación empírica se llevaron a cabo solamente en una clínica ambulatoria de Urología de un hospital especializado en Oncología, donde más de la mitad de la muestra estuvo conformada por hombres con cáncer. En consecuencia, los estudios a futuro deberán ampliar sus muestras para verificar la validez de los hallazgos en distintas regiones de Brasil y entre diferentes poblaciones dentro del campo de las enfermedades urológicas. Otra limitación consistió en que el estudio original no permitió realizar una comparación directa con la versión brasileña del instrumento, principalmente porque las variables utilizadas para validar la relación con variables externas se derivaron de exámenes de uroflujometría ^( 8 )^. La ausencia de datos de uroflujometría en las historias clínicas impidió replicar el análisis de evidencias del estudio original, lo que implicó comparar hallazgos con diarios de frecuencia-volumen y resultados de análisis de uroflujometría ^( 8 )^.

ICIQ-MLUTSsex-Versión brasileña aporta un importante valor teórico al proceso de adaptación transcultural de este instrumento. Esta versión se destaca por haber sido sometida a robustos análisis psicométricos, que confirmaron la presencia de una estructura interna que sustenta la interpretación de sus ítems. A diferencia de este enfoque integral, las adaptaciones para Sudáfrica, Canadá, Estados unidos, Corea, México y Eslovaquia fueron parte de un estudio multicéntrico con una muestra modesta compuesta por 30 participantes. Este estudio se enfocó primordialmente en los aspectos lingüísticos para garantizar que los ítems fueran fáciles de comprender ^( 9 )^, sin profundizar en ninguna verificación de otros tipos de evidencia de validez necesarios para medir el constructo con exactitud.

Utilizar este instrumento permitirá abrir nuevos horizontes interpretativos acerca de las DS en personas con síntomas STUI en Brasil. Sus resultados podrían sustentar el accionar asistencial de los enfermeros en distintos ámbitos de atención de la salud, al igual que medidas directas en pos de diseñar estrategias más efectivas para reducir el efecto de las DS sobre la calidad de vida de los hombres. Además, este trabajo de investigación aportar técnicas sólidas y se basó en recomendaciones psicométricas contemporáneas.

## Conclusión

Este estudio establece que ICIQ-MLUTSsex-Versión brasileña para hombres que padecen síntomas STUI (especialmente pacientes con cáncer) presenta evidencia sólida, tanto de validez como de fiabilidad. Eso señala un significativo avance porque se proporciona una vía de investigación novedosa para la evaluación inicial de las DS en brasileños con síntomas STUI, puesto que cubre el vacío dejado por la ausencia de otras herramientas validadas que vinculen los síntomas STUI con las DS. Se espera que esta medida PROM resulte extremadamente útil en próximos trabajos de investigación y en la práctica clínica.
